# Book Review

**Published:** 1906-09

**Authors:** 


**A Guide to the Administration of Ethyl Chloride.** By G. A. H. Barton, M.D. Pp. 36. London: H. K. Lewis. 1905.—This monograph probably owes its existence to the success which Dr. Barton has met with in his method of prolonging anæsthesia in intra-oral operations. It consists of standing a specially-devised bottle, containing ethyl chloride, in hot water and of delivering the vapour thus driven off into the patient's mouth by means of a rubber tube. Details of the method will be found on pp. 19 to 26. We should have welcomed a more exhaustive discussion on the merits of ethyl chloride from the point of view of safety. This is an important question, and the author has treated it somewhat loosely. In summing up he says: "Compared with other anæsthetics, I think there is no doubt that ethyl chloride comes some way after nitrous oxide gas in point of safety, but it is a good long way ahead of the rest." But this, we venture to think, is not in accordance with some authorities, who have recently assigned ethyl chloride a place between ether and chloroform.

**"First Aid" to the Injured and Sick: an advanced Ambulance Handbook.** By F. J. Warwick, M.B., and A. C. Tunstall, M.D. Fourth Edition. Pp. xiii., 242. Bristol: John Wright & Co. 1906.—We have in a former number of the *Journal* spoken of the usefulness of this book. It has been brought up to date. The additions to the chapters on digestion and excretion are a distinct advantage.

